# Structured Reporting of Computed Tomography in the Staging of Neuroendocrine Neoplasms: A Delphi Consensus Proposal

**DOI:** 10.3389/fendo.2021.748944

**Published:** 2021-11-30

**Authors:** Vincenza Granata, Francesca Coppola, Roberta Grassi, Roberta Fusco, Salvatore Tafuto, Francesco Izzo, Alfonso Reginelli, Nicola Maggialetti, Duccio Buccicardi, Barbara Frittoli, Marco Rengo, Chandra Bortolotto, Roberto Prost, Giorgia Viola Lacasella, Marco Montella, Eleonora Ciaghi, Francesco Bellifemine, Federica De Muzio, Ginevra Danti, Giulia Grazzini, Massimo De Filippo, Salvatore Cappabianca, Carmelo Barresi, Franco Iafrate, Luca Pio Stoppino, Andrea Laghi, Roberto Grassi, Luca Brunese, Emanuele Neri, Vittorio Miele, Lorenzo Faggioni

**Affiliations:** ^1^ Division of Radiology, “Istituto Nazionale Tumori IRCCS Fondazione Pascale – IRCCS di Napoli”, Naples, Italy; ^2^ Department of Radiology, IRCCS Azienda Ospedaliero-Universitaria di Bologna, Bologna, Italy; ^3^ Italian Society of Medical and Interventional Radiology (SIRM), SIRM Foundation, Milan, Italy; ^4^ Division of Radiology, “Università degli Studi della Campania Luigi Vanvitelli”, Naples, Italy; ^5^ Medical Oncology Division, Igea SpA, Napoli, Italy; ^6^ Medical Oncology Unit, Istituto Nazionale Tumori IRCCS ‘Fondazione G. Pascale’, Naples, Italy; ^7^ Department of Surgery, Istituto Nazionale Tumori -IRCCS- Fondazione G. Pascale, Naples, Italy; ^8^ Radiodiagnostics Section, DSMBNOS, “Aldo Moro” University, Bari, Italy; ^9^ Imaging Department, San Paolo Hospital, Savona, Italy; ^10^ Department of Radiology, Ospedali Civili, Hospital of Brescia, University of Brescia, Brescia, Italy; ^11^ Department of Radiological Sciences, Oncology and Pathology, Sapienza University of Rome - I.C.O.T. Hospital, Latina, Italy; ^12^ Department of Radiology, I.R.C.C.S. Policlinico San Matteo Foundation, Pavia, Italy; ^13^ Radiology Unit, Azienda Ospedaliera Brotzu, Cagliari, Italy; ^14^ Research and Development, Exprivia Spa, Bari, Italy; ^15^ Department of Medicine and Health Sciences “V. Tiberio”, University of Molise, Campobasso, Italy; ^16^ Division of Radiology, “Azienda Ospedaliera Universitaria Careggi”, Florence, Italy; ^17^ Department of Medicine and Surgery, Unit of Radiology, University of Parma, Maggiore Hospital, Parma, Italy; ^18^ Diagnostic Imaging Section, Department of Medical and Surgical Sciences & Neurosciences, Siena University Hospital, Siena, Italy; ^19^ Department of Radiological, Oncological and Pathological Sciences, Policlinico Umberto I, Sapienza University of Rome, Rome, Italy; ^20^ Radiology Department, University of Foggia, Foggia, Italy; ^21^ Department of Surgical and Medical Sciences and Translational Medicine, Sapienza University of Rome-Sant’Andrea University Hospital, Rome, Italy; ^22^ Department of Translational Research, University of Pisa, Pisa, Italy

**Keywords:** radiology report, structured report, staging, neuroendocrine neoplasm, computed tomography

## Abstract

**Background:**

Structured reporting (SR) in radiology is becoming increasingly necessary and has been recognized recently by major scientific societies. This study aims to build structured CT-based reports in Neuroendocrine Neoplasms during the staging phase in order to improve communication between the radiologist and members of multidisciplinary teams.

**Materials and Methods:**

A panel of expert radiologists, members of the Italian Society of Medical and Interventional Radiology, was established. A Modified Delphi process was used to develop the SR and to assess a level of agreement for all report sections. Cronbach’s alpha (Cα) correlation coefficient was used to assess internal consistency for each section and to measure quality analysis according to the average inter-item correlation.

**Results:**

The final SR version was built by including n=16 items in the “Patient Clinical Data” section, n=13 items in the “Clinical Evaluation” section, n=8 items in the “Imaging Protocol” section, and n=17 items in the “Report” section. Overall, 54 items were included in the final version of the SR. Both in the first and second round, all sections received more than a good rating: a mean value of 4.7 and range of 4.2-5.0 in the first round and a mean value 4.9 and range of 4.9-5 in the second round. In the first round, the Cα correlation coefficient was a poor 0.57: the overall mean score of the experts and the sum of scores for the structured report were 4.7 (range 1-5) and 728 (mean value 52.00 and standard deviation 2.83), respectively. In the second round, the Cα correlation coefficient was a good 0.82: the overall mean score of the experts and the sum of scores for the structured report were 4.9 (range 4-5) and 760 (mean value 54.29 and standard deviation 1.64), respectively.

**Conclusions:**

The present SR, based on a multi-round consensus-building Delphi exercise following in-depth discussion between expert radiologists in gastro-enteric and oncological imaging, derived from a multidisciplinary agreement between a radiologist, medical oncologist and surgeon in order to obtain the most appropriate communication tool for referring physicians.

## Introduction

Neuroendocrine neoplasms (NENs) are heterogeneous with respect to their site of origin and metastatic behaviour. About 25% of NENs secrete hormones leading to specific clinical signs and symptoms, while the remaining approximate 75% are so-called non-secreting NENs, which are often diagnosed incidentally and 40–50% are already in a metastatic tumour stage (1–3). Functioning NENs usually show up relatively early, so it might be difficult to detect lesions on radiological imaging, since these are often too small to be seen (4). While the detection and follow-up of NENs still pose a diagnostic challenge, radiological imaging is essential for the assessment of metastatic lesions (especially in the liver) and of tumour response to treatment, playing a key role in guiding treatment planning (5).

In such a complex scenario, an effective communication of imaging data to referring physicians is crucial for patient care. Radiology reports are the gold standard as to comprehensiveness and accuracy, and they are traditionally created as non-structured free text reporting (FTR) written in a narrative language. However, inconsistencies regarding content, style, and presentation format can reduce the clarity of FTR and hinder the communication of key information to the referring physician, potentially leading to incorrect diagnosis, delayed initiation of adequate treatment, or adverse patient outcomes (6–9).

Radiological structured reporting (SR) has several advantages over FTR, including a higher standardization of reporting style and lexicon with the adherence to established practice guidelines and recommendations, greater consistency and reproducibility (10–12), possibility of data mining and integration with artificial intelligence systems (13), shorter reporting time, lower error rate, and better communication with referring clinicians and other radiologists (8, 14, 15). To the latter regard, oncologists tend to prefer SR over FTR due to its ability to share information more clearly and in a more standardised way (16–18). Moreover, in 2018 the European Neuroendocrine Tumor Society (ENETS) launched an initiative to provide guidance for synoptic reporting of computed tomography (CT) and magnetic resonance imaging (MRI) examinations for staging and follow-up of bronchial, pancreatic and gastrointestinal NENs, where members of the imaging group stated a strong preference for a combination of limited and standardised options by way of drop-down menus, wherever possible (19).

Despite its established advantages, SR has not yet become commonplace in the radiological routine due to several reasons, including the current paucity of usable templates and of commercially available SR software solutions (6). In this context, the Italian Society of Medical and Interventional Radiology (SIRM) has created an Italian warehouse of SR templates that can be freely accessed by all SIRM members [including MRI for primary staging and restaging of rectal cancer (7), and chest CT in the management of COVID-19 pneumonia (9)], thus facilitating their routine use in a clinical setting (20).

Our purpose is to devise and evaluate an SR template for CT examinations performed for primary staging of NENs, with the goal to improve the standardization of reporting based on current best practice guidelines, as well as the communication between radiologists and clinicians, and among radiologists themselves.

## Materials and Methods

### Expert Panel

Following extensive discussion between expert radiologists, a multi-round consensus-building Delphi exercise was performed to develop a comprehensive, focused SR template for CT at the staging phase of patients with NENs.

A SIRM radiologist with experience in abdominal imaging created the first draft of the SR with the collaboration of a surgeon and medical oncologist specialised in NENs.

A working team of 14 expert radiologists was set up, with members from the SIRM Chapters of Gastrointestinal and Abdominal Radiology and of Diagnostic Imaging in Oncology. Their aim was to revise the initial draft iteratively, with the objective of reaching a final consensus on the SR.

### Selection of the Delphi Domains and Items

All panellists reviewed literature data on leading scientific databases (including PubMed, Scopus and Google Scholar), to assess papers on NENs, CT and structured radiology reports published from December 2000 to June 2021. The full text of the selected studies was reviewed by all members of the expert panel, and each of them developed and shared the list of Delphi items *via* email and/or teleconference.

The SR was divided into the following four sections: *(a)* Patient Clinical Data (including 16 items), *(b)* Clinical Evaluation (13 items), *(c)* Imaging Protocol (8 items) and *(d)* Report (17 items). A final section dedicated to key images was also included in the template.

Two Delphi rounds were performed. During the first round, each panellist independently contributed to refining the SR draft by means of online meetings or email exchanges. The level of panellists’ agreement for each SR model was tested in the second Delphi through a Google Form questionnaire shared by email. Each expert expressed individual comments for each specific template section by using a five-point Likert scale (1 = strongly disagree, 2 = slightly disagree, 3 = slightly agree; 4 = generally agree, 5 = strongly agree).

After the second Delphi round, the last version of the SR was generated on the dedicated RSNA website (www.radreport.org) by using a T-Rex template format, in line with IHE (Integrating Healthcare Enterprise) and MRRT (Management Of Radiology Report Templates) profiles and accessible as open-source software, with technical support by Exprivia (Exprivia SpA, Bari, Italy). Such profiles determine both the format of radiology report templates [using version 5 of Hypertext Markup Language (HTML5)] and the transporting mechanism to request, retrieve, and stock these schedules. The radiology report was structured by using a series of “codified queries” integrated in the T-Rex editor’s preselected sections (21).

### Statistical Analysis

Answers from each panellist were exported into Microsoft Excel^®^ format for ease of data collection and statistical analysis.

All panellists’ ratings for each section were analysed, with descriptive statistics measuring the mean score, the standard deviation, and the sum of scores. An average mark of 3 was considered good, whereas 4 was considered excellent.

To measure the internal consistency of panellist ratings for each section of the report, a quality analysis based on the average inter-item correlation was performed with Cronbach’s alpha (Cα) correlation coefficient (22, 23). The Cα test provides a measure of the internal consistency of a test or scale; it is expressed as a number between 0 and 1. Internal consistency describes the extent to which all the items in a test measure the same concept. Cα was determined after each round.

The nearer the Cα coefficient to 1.0, the more accurate the internal consistency of the categories in the scale. An alpha coefficient (α) ≥0.9 was considered excellent, α ≥0.8 good, α ≥0.7 acceptable, α ≥0.6 questionable, α ≥0.5 poor, and α <0.5 unacceptable. However, an α = 0.8 was seen as an acceptable parameter in internal reliability during iterations.

Data analysis was performed using Matlab Statistic Toolbox (The MathWorks, Inc., Natick, MA, USA).

## Results

### Structured Report

The final SR (**Appendix 1**) template was built by including n=16 items in the “Patient Clinical Data” section, n=13 items in the “Clinical Evaluation” section, n=8 items in the “Imaging Protocol” section, and n=17 items in the “Report” section. Overall, 54 items were included in the final version of the SR.

The “Patient Clinical Data” section included patient clinical data, previous or family history of malignancies, risk factors, endocrine and or neuroendocrine neoplasms in young age, hereditary syndromes, and other genetic mutations. In this section, we included the item “Allergies” to the drug or no drug and contrast medium.

The “Clinical Evaluation” section collected previous examination results, a genetic panel, results of histopathological examination on biopsy specimen, Chromogranin A (CgA) level, Neuron-specific enolase (NSE) level, 5-hydroxyindoleacetic acid (5-HIAA) 24-h urine level, serum gastrin level, serum insulin level, serum glucagon level, serum VIP level, blood count, serum creatinine, liver function and clinical symptoms.

The “Imaging Protocol” section included data on the equipment used, the number of detectors and whether it was multidetector or dual energy, including data on the reconstruction algorithm and slice thickness. In addition, we collected data on contrast study protocol, including data on the contrast study phase, as well as data concerning the contrast medium, such as the active principle, commercial name, dosage, flow rate, concentration, and ongoing adverse events. In addition, in this section we included data about bowel preparation and contrast technique for gastro-enteric tract evaluation.

The “Report” section included data on lesion sites (primary tumour visible or not visible on CT imaging) and features such as number, site, size, and infiltration of neighbouring organs and/or structures for each site (e.g., lung, gastric, pancreatic, duodenal, small bowel, appendiceal and colorectal lesion). For small bowel lesions, we evaluated the desmoplastic reaction, while for pancreatic lesions assessment was made on the relationship with the pancreatic duct, with vessels and duodenum or ampulla, according to surgical planning. In addition, in this section we included tumour stage, node stage and metastases stage, as well as the presence of incidental radiological findings. To allow for maximum flexibility of SR use in different working scenarios, only the Report section fields are mandatory, whereas all fields from the other SR sections can be filled in upon user discretion.

### Consensus Agreement

**Tables 1**, **2** show single scores and the sum of scores of the 14 panellists for the SR in the first and second rounds, respectively.

**Table 1:** Single scores and sum of scores of 14 panellists for the structured report in the first round.

Panellist #	A1. Anthropometric data	A2. Personal assessments	A3. Allergies and adverse reactions	B1. Clinical information	C1. Exam data	C2. Contrast agent	C4. Adverse Events	D1. Lesion	D2. Lymphadenopathy	D3. Distantmetastasis	D8. Accessoryfindings	Total
1	5	4	5	5	5	5	5	5	5	5	5	54
2	5	4	5	5	5	5	5	5	5	5	5	54
3	3	3	5	5	5	5	5	5	5	5	5	51
4	4	4	4	5	5	5	5	5	5	5	4	51
5	5	5	5	5	5	5	5	5	5	5	5	55
6	5	5	5	5	4	5	5	5	5	5	5	54
7	5	5	5	5	5	5	5	5	5	5	5	55
8	4	4	5	4	5	5	5	4	5	5	5	51
9	5	5	5	5	5	5	5	5	5	5	1	51
10	4	4	5	5	5	5	5	5	5	5	5	53
11	5	5	5	5	5	5	5	5	5	5	4	54
12	3	4	5	5	5	5	5	5	5	3	3	48
13	5	3	4	5	3	5	4	3	5	3	5	45
14	4	4	5	5	5	5	5	5	5	5	4	52
Mean	4.43	4.21	4.86	4.93	4.79	5.00	4.93	4.79	5.00	4.71	4.36	52.00
Std	0.73	0.67	0.35	0.26	0.56	0.00	0.26	0.56	0.00	0.70	1.11	2.83

**Table 2:** Single scores and sum of scores of 14 panellists for the structured report in the second round.

Panellist #	A1. Anthropometric data	A2. Personal assessments	A3. Allergies and adverse reactions	B1. Clinical information	C1. Exam data	C2. Contrast agent	C4. Adverse Events	D1. Lesion	D2. Lymphadenopathy	D3. Distant metastasis	D8. Accessory findings	Total
1	5	5	5	5	5	5	5	5	5	5	5	55
2	5	5	5	5	5	5	5	5	5	5	5	55
3	5	5	5	5	5	5	5	5	5	5	5	55
4	5	5	5	5	5	5	5	5	5	5	5	55
5	5	5	5	5	5	5	5	5	5	5	4	54
6	5	5	5	5	5	5	5	5	5	5	5	55
7	4	4	4	5	5	5	5	5	5	5	4	51
8	5	5	5	5	5	5	5	5	5	5	5	55
9	5	5	5	5	5	5	5	5	5	5	5	55
10	5	5	5	5	5	5	5	5	5	5	5	55
11	5	5	5	5	5	5	5	5	5	5	5	55
12	5	4	4	5	4	4	4	5	5	5	5	50
13	5	5	5	5	5	5	5	5	5	5	5	55
14	5	5	5	5	5	5	5	5	5	5	5	55
Mean	4.93	4.86	4.86	5.00	4.93	4.93	4.93	5.00	5.00	5.00	4.86	54.29
Std	0.27	0.36	0.36	0.00	0.27	0.27	0.27	0.00	0.00	0.00	0.36	1.64

Both in the first and second round, all sections received more than a good rating, with a mean value of 4.7 (range 4.2-5.0) in the first round, and a mean value of 4.9 (range 4.9-5) in the second round.

In the first round, the Cα correlation coefficient was 0.57, with an overall mean score of experts and sum of scores for the SR being 4.7 (range 1-5) and 728 (mean value 52.00, standard deviation 2.83), respectively.

In the second round, the Cα correlation coefficient was 0.82, with an overall mean score of experts and sum of scores for the SR being 4.9 (range 4-5) and 760 (mean value 54.29, standard deviation 1.64), respectively.

## Discussion

To the best of our knowledge, this is the first time that a panel of experts from a national radiological society has promoted the creation of a comprehensive SR template for the CT staging of NENs, systematically encompassing all steps of the radiological procedure (from registration of patient personal data to full details of CT examination and standardised reporting of relevant CT findings), along with the possibility of coupling together radiological and clinical data and in the perspective of integrating the SR template into the radiology workflow.

Our SR template was based on a multi-round consensus-building Delphi exercise following in-depth discussion between expert radiologists in gastrointestinal and oncological imaging. On a previous occasion, panellists had assessed and promoted a SR for MRI-based primary staging and restaging of rectal cancer (7). While such MRI templates were also based on a multi-round consensus-building Delphi exercise, in that earlier study the original draft derived from a single dedicated radiologist, without seeking any multidisciplinary agreement. On the other hand, in the present study the SR draft wa based on the multidisciplinary agreement of a radiologist, a medical oncologist and a surgeon, with the goal to achieve an optimal, all-around communication tool for referring physicians.

The approved SR template was divided into four sections (i.e., Patient Clinical Data, Clinical Evaluation, Imaging Protocol and Report), with a final section dedicated to key images. Although our template may appear somewhat long and complex (potentially slowing down the radiology workflow), it must be emphasised that only the Report section is mandatory, whereas other sections are optional. Furthermore, considering that not all data may be available to the radiologist, these open fields can also be filled in at a subsequent time. In addition, the possibility of connecting this template with the patient electronic health record allows for automatic import of available data.

All sections received more than a good rating in both Delphi rounds, with a mean value of 4.7 and 4.9 in the first and in the second round, respectively. In the first round, the Cα correlation coefficient was relatively poor (0.57), whereas in the second round, it was substantially improved (0.82). Moreover, the overall mean score of the experts in the second round was higher than that of the first round, with a lower standard deviation value being related to a greater agreement among experts for this SR. The sections with the lowest level of agreement were “Patient Clinical Data” and “Clinical Evaluation”, reflecting the opinion that these sections were too long and may slow down daily practice. However, following a conference call, all panellists expressed their agreement once the optional nature of the sections had been clarified, and the importance of collecting patient clinical data and history for big data creation and connecting radiological with clinical data was demonstrated as well.

The “Patient Clinical Data” section included patient data, previous or family history of malignancies, risk factors, endocrine and or neuroendocrine neoplasms in young age, hereditary syndromes, and other genetic mutations. The “Clinical Evaluation” section collected previous examinations findings, a genetic panel, results of histopathological examination on biopsy specimen, chromogranin A (CgA) level, neuron-specific enolase (NSE) level, 5-hydroxyindoleacetic acid (5-HIAA) 24-h urine level, serum gastrin level, serum insulin level, serum glucagon level, serum VIP level, blood count, serum creatinine, liver function and clinical symptoms. This data could serve as a basis for creating potentially large databases, allowing not only for epidemiological statistical analysis, but also for building a radiomics model through the combination of radiological features and clinical data (24–29). To this end, genomic data could also be leveraged to build a radiogenomics model, which may be useful in the upper levels of personalised risk stratification and advanced precision medicine for early cancer diagnosis, cancer therapy selection, prognosis prediction, and assessment of treatment response and resistance to therapy (30–34).

A “strong agreement” was the result for the “Imaging Protocol” and “Report” sections. The “Imaging Protocol” section included data on the CT equipment used and related technical parameters (e.g., number of detector rows, multidetector single-or dual-energy, reconstruction algorithm and slice thickness). In addition, this section included data regarding the CT acquisition protocol (including post-contrast phases) and contrast medium administration (such as the active principle, commercial name, volume, flow rate, concentration, and ongoing adverse events), as well as data about bowel preparation and contrast technique for the evaluation of the gastrointestinal tract. Sharing examination techniques (not only within one’s own department, but also with the radiology departments of other centres) allows for the standardisation and optimisation of study protocols. Indeed, during the follow-up phase, differences in acquisition parameters and segmentation algorithms are significant features that can lead to variability in volumetric assessment. Thus, slice thickness and other protocol-related factors (such as the reconstruction kernel and field of view) should remain constant for reliable measurements to be performed. In the protocol optimization stage, enhanced communication between different centres can lead to an overall quality improvement through optimization of radiation dose and contrast administration, higher patient safety and overall better diagnostic quality (35, 36). Moreover, such enhanced communication can facilitate comparing the results obtained from different studies, thus reducing the variability due to different imaging protocols (37, 38).

The “Report” section included data on primary tumour being visible or not on CT imaging, as well as features such as lesion site, number, size, and infiltration of adjacent organs and/or structures for each site (e.g., lung, gastric, pancreatic, duodenal, small bowel, appendiceal, and colorectal lesions). Desmoplastic reaction was evaluated for small bowel lesions, whereas for pancreatic lesions the relationship with the pancreatic duct, vessels and duodenum or ampulla was taken into account, according to prospective surgical planning. In addition, in this section we included tumour stage, node stage and metastases stage, as well as the presence of incidental radiological findings. The possibility of using a SR template to guide the radiology workflow allows describing all the main radiological features that could be omitted with FTR, e.g., by mere distraction. For example, in pancreatic NENs, a correct evaluation of the stage of the neoplasm and an assessment of the relationship with the pancreatic duct, vessels, duodenum or the infiltration of neighbouring structures, allows for a correct stratification of patients and can avoid unnecessary major surgery compared to tumour enucleation (39–42). Using a checklist and a systematic search pattern may help to prevent such diagnostic errors. Both radiologists and referring clinicians are keen to reduce the rate of diagnostic errors, which, for radiologists, accounts for as much as 4% of reports (43–47). A retrospective overview of 3,000 MRI examinations was useful in determining clinically relevant extraspinal results in 28.5% of patients, which were not present in initial, unstructured reports (48). Similarly, the use of a checklist-style SR template has been shown to improve the rate of diagnosis of fracture-unrelated findings on cervical CT (49). SRs have also been shown to enhance the clinical impact on tumour staging and surgical planning for pancreatic and rectal carcinoma (50–52). Brook et al. compared the results of structured versus nonstructured reporting of CT findings for the staging and assessment of resectability for pancreatic cancer, and they concluded that surgeons were more confident about tumour resectability using SR compared to FTR (50). Sahni et al. showed that the use of an MRI-based SR improved rectal cancer staging when compared to FTR (52).

According to Weiss et al. [who described three levels of SR (53)], our SR is based on standardised terminology and structure, which are features needed for adhering to diagnostic-therapeutic recommendations and enrolment in clinical trials. This also reduces any ambiguity that may arise from non-conventional language and enables better communication between radiologists and clinicians. In addition, this SR template has the advantage of having been validated by a multidisciplinary group, potentially helping radiologists provide referring clinicians with all data required for correct patient management. However, this study has several limitations. Firstly, panellists were of the same nationality, and the contribution of experts from multiple countries could have allowed for a broader sharing, potentially increasing the consistency of the SR. Secondly, our study was not aimed at assessing the impact of the SR on the clinical management of NEN patients.

## Conclusion

We developed a SR template for primary CT staging of NENs based on a multi-round consensus-building Delphi exercise, following in-depth discussion between expert radiologists in gastrointestinal and oncological imaging and derived from the multidisciplinary agreement of a radiologists, a medical oncologist and a surgeon specialised in NENs. A widespread adoption of SR could improve the quality, clarity, and reproducibility of reports across hospital departments and locations, improving patient health care and fostering research development.

## Data Availability Statement

The original contributions presented in the study are included in the article/**Supplementary Material**. Further inquiries can be directed to the corresponding author.

## Ethics Statement

The studies involving human participants were reviewed and approved by Protocol number is: 13261–OSS, Azienda Ospedaliero - Universitaria Careggi, Florence, Italy. The patients/participants provided their written informed consent to participate in this study.

## Author Contributions

All authors listed have made a substantial, direct, and intellectual contribution to the work, and approved it for publication.

## Conflict of Interest

The authors declare that the research was conducted in the absence of any commercial or financial relationships that could be construed as a potential conflict of interest.

## Publisher’s Note

All claims expressed in this article are solely those of the authors and do not necessarily represent those of their affiliated organizations, or those of the publisher, the editors and the reviewers. Any product that may be evaluated in this article, or claim that may be made by its manufacturer, is not guaranteed or endorsed by the publisher.
