# Supplementary material for: Structured Reporting of Computed Tomography in the Staging of Neuroendocrine Neoplasms: A Delphi Consensus Proposal
Source: Front Endocrinol (Lausanne). 2021 Nov 30;12:748944. doi: 10.3389/fendo.2021.748944 (PMC8670531; doi:10.3389/fendo.2021.748944)
Supplement: Supplementary file 1 [file DataSheet_1.docx]

**PATIENT’S CLINICAL DATA (*imported from RIS)**

| FIELD | DETAIL | ADMITTED VALUES | | |
| --- | --- | --- | --- | --- |
| ANTHROPOMETRIC DATA | | | | |
| Weight |  | (kg) *[Numeric]* | | |
| Height |  | (cm) *[Numeric]* | | |
| BMI |  | *[Numeric]* (automatically calculated) | | |
| BSA |  | *[Numeric]* (automatically calculated) | | |
| Age |  | (years) *[Numeric]* | | |
| Age range |  | - < 50 years - > 50 years | | |
| PATIENT HISTORY | | | | |
|  |  | |  |  |
| Family history of cancer  (visible only if “Yes” and repeatable) | Yes/No |  | | |
|  | Degree of kinship | - Mother - Father - Brother(s)/sister(s) - Maternal grandparent(s) - Paternal grandparent(s) - Uncle(s)/aunt(s) - Other *[free text]* | | |
|  | Notes | *[free text]* | | |
| Personal history of other cancers | Yes/No |  | | |
|  | Notes | *[free text]* | | |
| Endocrine/neuroendocrine neoplasms in young age  (visible only if “Yes” and repeatable) | Yes/No |  | | |
|  | Type | - Pituitary gland - Breast - Ovary - Thyroid - Parathyroid - Adrenal gland - Pancreas | | |
|  | Notes | *[free text]* | | |
| Hereditary syndromes | Yes/No | - Neurofibromatosis type I - Tuberous sclerosis - MEN1 - MEN2 - Von Hippel-Lindau | | |
| Other genetic mutations | Yes/No | if yes, specify *[free text]* | | |
| Lifestyle/dietary habits  (visible only if “Yes” and repeatable) | Smoker | Yes/No | | |
|  | SMOKING DETAILS (visible only if Smoke = “Yes”) | | | |
|  |  | Smoker | | - Current smoker - Former smoker |
|  |  | Cigarette smoke | | Yes/No |
|  |  | Number of daily cigarettes (if “Current smoker”) | | - Light (< 15) - Heavy (≥15) |
|  |  | Years of smoking | | *[Numeric]* |
|  |  | Years from cessation  (if “Former smoker”) | | - ≤ 15 - >15 |
|  |  | Cigarettes per year  (packs/year)  (if “Former smoker” or “Current smoker”) | | *[Numeric]* (automatically calculated)*  *(Number of daily cigarettes x smoking years / 20) |
|  |  | Vaping | | Yes/No |
|  |  | Number of daily electronic cigarette refills (if vaping = “Yes”) | | *[Numeric]* |
|  |  | Number of years  (if vaping = “Yes”) | | *[Numeric]* |
|  |  | Notes *[free text]* | |  |
|  |  |  | | |
|  | High alcohol intake | Yes* *(more than 1 glass/day for women and 2 glasses/day for men)  No | | |
|  | High meat intake | Yes* *(white or red meat intake more than 3 times/week)  No | | |
|  | High cured meat intake | Yes *(cured meat intake more than once a week)  No | | |
|  | Low vegetable intake | Yes *(less than 2 servings/day)  No | | |
|  | Low fruit intake | Yes (*less than 3 whole fruits/day)  No | | |
|  | Notes | *[free text]* | | |
| ALLERGIES AND ADVERSE REACTIONS | | | | |
| Reported allergies  (visible only if “Yes” and repeatable) | Yes/No |  | | |
|  | Type | - Drug-related (n of drugs) - Contrast medium-related (n of contrast media) - Drug-unrelated | | |
|  | Active principle/molecule  [if drug- or contrast medium-related allergy] | *[free text]* | | |
|  | Commercial name  [if drug- or contrast medium-related allergy] | *[free text]* | | |
|  | Notes | *[free text]* | | |
| PREVIOUS adverse reactions  (visible only if “Yes” and repeatable) | Yes/No |  | | |
|  | Date | month/year [mm/yyyy] | | |
|  | Type | Contrast medium-related / unrelated | | |
|  | Degree | - Mild - Moderate - Severe | | |
|  | Time of onset | - Early - Late | | |
|  | Notes | *[free text]* | | |
| Antiallergic premedication | Yes/No |  | | |
|  | Treatment | - Steroid - Antihistamine | | |
|  | Complete | Yes/No | | |
|  | Notes | *[free text]* | | |
| Nephroprotective protocol | Yes/No |  | | |
|  | Complete | Yes/No | | |
|  | Serum creatinine | *[Numeric]* (mg/dl) | | |
|  | GFR (Glomerular Filtration Rate) | *[Numeric]* (ml/min)  <https://www.merckmanuals.com/medical-calculators/GFR_CKD_EPI-it.htm> (gender, race, age, serum creatinine) | | |
|  | Notes | *[free text]* | | |

**CLINICAL EVALUATION**

| FIELD | DETAIL | ADMITTED VALUES |
| --- | --- | --- |
| CLINICAL INFORMATION | | |
| Prior examinations  (visible only if “Yes” and repeatable) | Yes/No |  |
|  | Type | - Colonoscopy - Gastroscopy - Bronchoscopy - Mediastinoscopy - Videothoracoscopy - CT - MRI - US - 18F-FDG PET/CT - 68-Ga-DOTA-labeled PET/CT - 18F-Dopa PET/CT con 18F-Dopa - 111In-pentetreotide scintigraphy - SRS Octreoscan - SPECT/CT - Other *[free text]* |
|  | Date | *[dd/mm/yyyy]* |
|  | Notes | *[free text]* |
| Histopathological examination on biopsy specimen | Yes/No |  |
|  | Chromogranin | +/- |
|  | Synaptophysin | +/- |
|  | CD56 | +/- |
|  | Histologic subtype  NET G1  NET G2  NET G3 | (specify)  Yes/No  Yes/No  Yes/No |
|  | NEC | Yes/No |
| Chromogranin A (CgA) level |  | *[Numeric]* |
| Neuron-specific enolase (NSE) level |  | *[Numeric]* |
| 5-hydroxyindoleacetic acid (5-​HIAA) 24-h urine level |  | *[Numeric]* |
| Serum gastrin level |  | *[Numeric]* |
| Serum insulin level |  | *[Numeric]* |
| Serum glucagon level |  | *[Numeric]* |
| Serum VIP level |  | *[Numeric]* |
| Blood count |  | *[Numeric]* |
| Serum creatinine |  | *[Numeric]* |
| Liver function |  | - Normal - Impaired |
| Symptomatic | Yes/No | If yes:   - Disabling diarrhoea - Rash with face and neck flushing - Tachycardia - Hyperhidrosis - Visual disturbances |

**IMAGING PROTOCOL**

| FIELD | DETAIL | ADMITTED VALUES |
| --- | --- | --- |
| IMAGING DATA | | |
| Date of examination |  | Date *[dd/mm/yyyy]* |
| Clinical indication | Primary staging |  |
| Scanner brand and model |  | *[free text]* |
| Scanning technique | Number of detector rows | *[Numeric]* |
|  | Precontrast scan  ***(details visible only if “Yes”)*** | Yes/No  Dual energy (Yes/No)  Slice thickness (mm) *[Numeric]*  Convolution kernel(s) *[free text]*  Body area *[multiple choice]*:   - abdomen - chest - neck - brain |
|  | Post-contrast scan  ***(details repeatable for each post-contrast scan)*** | *[Numeric]*  Post-contrast phase(s) (arterial, venous, late)  Dual energy (Yes/No)  Slice thickness (mm) *[Numeric]*  Convolution kernel(s) *[free text]*  Body area *[multiple choice]*:   - abdomen - chest - neck - brain |
| Bowel preparation | Yes/No | If yes, specify *[free text]* |
| Radiation exposure | Class of radiation exposure | *[Numeric]* |
| CONTRAST MEDIUM | | |
| Use of contrast medium  (visible only if “Yes”) | Yes/No |  |
|  | Active principle | - Iobitridol - Iodixanol - Iohexol - Iomeprol - Iopromide - Ioversol |
|  | Commercial name | *[free text]* |
|  | Volume | *[Numeric]* (ml) |
|  | Flow rate | *[Numeric]* (ml/sec) |
|  | Concentration | *[Numeric]* (mg I/ml) |
|  | Notes | *[free text]* |
| Contrast technique for GI tract evaluation | - Double contrast, stomach - Enteroclysis - Enterography | Specify contrast medium used for GI tract distention:   - type *[free text]* - amount (ml) *[Numeric]* |
| ADVERSE EVENTS | | |
| ONGOING adverse events  (visible only if “Yes”) | Yes/No |  |
|  | Date and hour of event | *[dd/mm/yyyy, hour]* |
|  | Degree | - Mild - Moderate - Severe |
|  | Time of onset | - Early - Late   Minutes *[Numeric]* (optional) |
|  | Type | ALLERGIC / ALLERGIC-LIKE  **Mild**   - Sparse wheals/itch - Skin oedema - Mild itching / feeling like ”velvet in the throat“ - Nasal congestion - Sneezing - Conjunctivitis - Rhinorrhoea   **Moderate**   - Diffuse wheals/intense itch - Diffuse skin oedema - Facial oedema without dyspnea - Feeling of choking or hoarseness - Wheezing / mild bronchospasm without hypoxia   **Severe**   - Dyspnea - Erythema – diffuse mucocutaneous symptoms - Laryngeal oedema with stridor and/or hypoxia - Wheezing / bronchospasm - Significant hypoxia - Anaphylactic shock (severe hypotension and brady-tachyarrhythmia)   NON-ALLERGIC  **Mild**   - Mild nausea/limited vomiting - Transient chills / heat / redness - Headache / dizziness / anxiety / altered taste - Slight increase in blood pressure - Self-limiting vasovagal reaction   **Moderate**   - Prolonged nausea/vomiting - Elevated arterial blood pressure - Isolated chest pain - Vasovagal reaction   **Severe**   - Treatment-refractory vasovagal reaction - Arrhythmia - Convulsions - Severe arterial hypertension   CONTRAST MEDIUM EXTRAVASATION |
|  | Type of treatment | - Wait and see - Drug therapy (specify in “Notes” field) - Anaesthesiologist’s intervention required |
|  | Event resolution | - Spontaneous - After treatment - After hospitalisation - Other [free text] |
|  | Notes | *[free text]* |

**REPORT**

| FIELD | DETAIL | | ADMITTED VALUES |
| --- | --- | --- | --- |
| DIAGNOSIS | | | |
| PRIMARY TUMOUR  (open following fields depending on the “Site” of primary tumour) | | | |
|  | | Lesion  ****note: primary tumour visible or not visible on CT imaging*** | - Visible* - Not visible* |
|  |  | Notes | *[free text]* |
|  |  | Site | - Lung - Stomach - Pancreas - Small bowel - Appendix - Colon-rectum |
| Lung lesions  **repeat subfields after “Number” for each of the N lesions* | | Number  Site  Size  Infiltration of neighbouring structures | *[Numeric]**   - Right  1. RUL 2. ML 3. RLL  - Left  1. LUL 2. LLL   Maximum diameter on axial images (mm) *[Numeric]*  Diameter perpendicular to maximum diameter (mm) *[Numeric]*  Yes/No  Specify *[free text]* |
| Gastric lesions  **repeat subfields after “Number” for each of the N lesions* | | Number  Site  Size  Limited to stomach wall  Extraparietal involvement  Infiltration of neighbouring structures | *[Numeric]**  Fundus  Body  Antrum  mm *[Numeric]*  Yes/No  Yes/No  Yes/No  Specify *[free text]* |
| Pancreatic lesions  **repeat subfields after “Number” for each of the N lesions* | | Number  Site  Size  Relationship with pancreatic duct  Relationship with vessels  Relationship with duodenum / ampulla  Infiltration of neighbouring structures | *[Numeric]**   - Head - Uncinate process - Body - Tail   mm *[Numeric]*  Yes/No  Yes/No  Yes/No  Yes/No  Specify *[free text]* |
| Duodenal / ampullar lesions  **repeat subfields after “Number” for each of the N lesions* | | Number  Site  Size  Sphincter of Oddi infiltration  Pancreatic infiltration  Peritoneal infiltration and/or of neighbouring organs | *[Numeric]**  Specify *[free text]*  mm *[Numeric]*  Yes/No  Yes/No  Yes/No  Specify *[free text]* |
| Small bowel lesions  **repeat subfields after “Number” for each of the N lesions* | | Number  Site  Size  Limited to bowel wall  Extraparietal growth  Infiltration of neighbouring organs and/or structures  Desmoplastic reaction  Other | *[Numeric]**   - Proximal jejunum - Distal jejunum - Proximal ileum - Distal ileum   mm *[Numeric]*  Yes/No  Yes/No  Yes/No; if yes, specify *[free text]*  Yes/No  *[free text]* |
| Appendiceal lesions | | Size  Limited to bowel wall  Extra-appendiceal growth  Infiltration of neighbouring organs and/or structures | mm *[Numeric]*  Yes/No  Yes/No  Yes/No  if yes, specify *[free text]* |
| Colorectal lesions  **repeat subfields after “Number” for each of the N lesions* | | Number  Site  Size  Infiltration of neighbouring organs and/or structures  Other | *[Numeric]**   - Caecum/ascending colon - Hepatic flexure - Proximal transverse colon - Distal transverse colon - Splenic flexure - Descending colon - Sigmoid colon - Rectum   mm *[Numeric]*  Yes/No  if yes, specify *[free text]*  *[free text]* |

| Lymph node metastases |  |
| --- | --- |
| Loco-regional | Yes/No  if yes, specify:   - number *[Numeric]* - site *[free text]* |
| Distant metastases  *(open following fields if “Yes”)* | Yes/No |
| Liver | Yes/No  if yes, specify:  Number of visible lesions *[Numeric]*  For each target lesion (max N=2):   - site *[Liver segment]* - maximum diameter on axial images (mm) *[Numeric]* - diameter perpendicular to maximum diameter (mm) *[Numeric]* - Structure (isovascular/hypovascular/hypervascular) |
| Lung | Yes/No  if yes, specify:  Number of visible lesions *[Numeric]*  For each target lesion (max N=2):   - site *[Lung lobe, Lung segment]* - maximum diameter on axial images (mm) *[Numeric]* - diameter perpendicular to maximum diameter (mm) *[Numeric]* |
| Non-regional lymph nodes | Yes/No  if yes, specify:   - site *[free text]* - maximum diameter on axial images (mm) *[Numeric]* - diameter perpendicular to maximum diameter (mm) *[Numeric]* |
| Other organs (incl. skeleton) | Yes/No  if yes, specify:  Site and type *[free text]* |

| Incidental findings |  |  |
| --- | --- | --- |
|  | Brain | *[free text]* |
|  | Neck | *[free text]* |
|  | Chest | *[free text]* |
|  | Abdomen | *[free text]* |
|  | Other | *[free text]* |

| CONCLUSIONS | *[free text]* |
| --- | --- |

**IMAGES**

| FIELD | DETAIL | ADMITTED VALUES |
| --- | --- | --- |
| Key images | Key images | *[Image]* |
